# A multicenter, prospective, non‐interventional real‐world study to assess the effectiveness of mecapegfilgrastim in preventing neutropenia in patients with gastrointestinal cancer

**DOI:** 10.1002/iid3.1348

**Published:** 2024-08-06

**Authors:** Chenyu Mao, Ye He, Nong Xu, Haijiao Yan, Ningling Zhang, Gang Cheng, Hua Jiang, Minbin Chen, Yong Chen, Xiaoguang Wang, Yulan Gu, Peng Shen, Guifang Zhang, Jun Yan, Zhe Yang, Lifang Ding, Zhengxiang Han, Zhanggui Wang, Junqi Zhang, Weie Zheng, Jufeng Wang, Shukui Qin

**Affiliations:** ^1^ Department of Medical Oncology, The First Affiliated Hospital, College of Medicine Zhejiang University Hangzhou China; ^2^ Department of Medical Oncology, Sun Yat‐sen University Cancer Center, State Key Laboratory of Oncology in South China, Guangdong Provincial Clinical Research Center for Cancer Sun Yat‐sen University Guangzhou China; ^3^ Department of Oncology The First People's Hospital of Changzhou Changzhou China; ^4^ Department of Oncology Affiliated Hospital of North Sichuan Medical College Nanchong China; ^5^ Department of Oncology Bozhou People's Hospital Bozhou China; ^6^ Department of Oncology The Second People's Hospital of Changzho Changzhou China; ^7^ Department of Oncology The First People's Hospital of Kunshan Kunshan China; ^8^ Department of Radiology, The First Affiliated Hospital Sun Yat‐sen University Guangzhou China; ^9^ Department of Hepatobiliary and Pancreatic Surgery The Second Hospital of Jiaxing Jiaxing China; ^10^ Department of Oncology Changshu No 2 People's Hospital Changshu China; ^11^ Department of Medical Oncology Xinxiang Central Hospital Xinxiang China; ^12^ Department of Oncology The Central Hospital of Jiading Shanghai China; ^13^ Department of Radiology Shandong Provincial Hospital Jinan China; ^14^ Department of Oncology The People's Hospital of Danyang Danyang China; ^15^ Depatment of Integrated Traditional Chinese and Western Medicine The Affiliated Hospital of Xuzhou Medical University Xuzhou China; ^16^ Department of Radiology The Second People's Hospital of Anhui Province Hefei China; ^17^ Department of Oncology The Central Hospital of Bazhong Bazhong China; ^18^ Department of Medical Oncology The People's Hospital of Rui'an Rui'an China; ^19^ Depatment of Gastroenterology Henan Cancer Hospital Zhengzhou China; ^20^ Chief of Hospital Nanjing Tianyinshan Hospital Nanjing China

**Keywords:** gastrointestinal, mecapegfilgrastim, neutropenia, real‐world

## Abstract

**Background:**

Mecapegfilgrastim, a long‐acting granulocyte‐colony stimulating factor has been approved for reducing the incidence of infection, particularly febrile neutropenia (FN), in China.

**Objective:**

We conducted a multicenter prospective observational study to examine the safety and effectiveness of mecapegfilgrastim in preventing neutropenia in gastrointestinal patients receiving the chemotherapy, including S‐1/capecitabine‐based regimens or the fluorouracil, leucovorin, oxaliplatin, and irinotecan (FOLFOXIRI)/fluorouracil, leucovorin, and oxaliplatin (FOLFOX)/fluorouracil, leucovorin, oxaliplatin, and irinotecan (FOLFIRINOX) regimens.

**Method:**

Five hundred and sixty‐one gastrointestinal patients from 40 sites across China, between May 2019 and November 2021, were included. The administration of mecapegfilgrastim was prescribed at the discretion of local physicians.

**Results:**

The most common adverse drug reactions (ADRs) of any grade for all patients was increased white blood cells (2.9%). Grade 3/4 ADRs were observed for anemia (0.2%), decreased white blood cells (0.2%), and decreased neutrophil count (0.2%). Among the 116 patients who received S‐1/capecitabine‐based chemotherapy throughout all cycles, ADRs of any grade included anemia (1.7%), myalgia (0.9%), and increased alanine aminotransferase (0.9%). No grade 3/4 ADRs were observed. In 414 cycles of patients who underwent S‐1/capecitabine‐based regimens, only one (0.2%) cycle experienced grade 4 neutropenia. In the FOLFIRINOX, FOLFOXIRI, and FOLFOX chemotherapy regimens, grade 4 neutropenia occurred in one (2.7%) of 37 cycles, four (4.7%) of 85 cycles, and two (1.2%) of 167 cycles, respectively.

**Conclusion:**

In a real‐world setting, mecapegfilgrastim has proven effective in preventing severe neutropenia in gastrointestinal patients following chemotherapy. This includes commonly used moderate or high‐risk FN regimens or regimens containing S1/capecitabine, all of which have demonstrated favorable efficacy and safety profiles.

## 1 INTRODUCTION

Gastrointestinal cancer, which includes cancers of the stomach, liver, esophagus, pancreas, and colorectum, accounted for an estimated 4.8 million new cases and 3.2 million deaths worldwide in 2022, as reported by the GLOBOCAN database.^1^ It represents a primary medical and economic burden globally and consistently has the highest number of new cancer cases and deaths annually. Chemotherapy stands as a common treatment regimen for gastrointestinal cancer.^2^, ^3^, ^4^, ^5^ However, it often leads to neutropenia, a frequent and dose‐limiting toxicity for patients, representing the most severe hematological toxicity of myelosuppressive chemotherapy.^6^ Chemotherapy‐induced neutropenia refers to the reduction in absolute neutrophil count (ANC) in peripheral blood following the use of myelosuppressive chemotherapeutic agents.^7^ Febrile neutropenia (FN) is a serious clinical complication that can result in dose reductions or treatment delays in chemotherapy, thereby reducing clinical efficacy. It can also lead to serious complications such as severe infections, and even death. The severity and duration of neutropenia are directly associated with the risk of infection and death, significantly affecting the relative dose intensity and established cycles of chemotherapy, making it challenging to achieve the desired treatment outcomes.^8^, ^9^ Therefore, prevention or treatment of neutropenia is essential to ensure adequate dosing or dose‐dense chemotherapy.

In 2002, the introduction of pegylated filgrastim (pegfilgrastim), the first long‐acting recombinant human granulocyte‐colony stimulating factor (rhG‐CSF), revolutionized the landscape of supportive care for chemotherapy‐induced neutropenia. Compared to short‐acting G‐CSFs, which require daily injections until neutrophil recovery due to rapid kidney clearance, long‐acting G‐CSFs have been modified to increase molecular size and thus evade kidney clearance, with their primary clearance from the body occurring through circulating neutrophils. Therefore, during neutropenia, serum levels of long‐acting G‐CSFs remain elevated, but they decrease as neutrophil levels return to normal.^10^ The long‐acting G‐CSFs provided a simplified approach by offering a once‐per‐chemotherapy‐cycle option.^11^, ^12^ Since then, there have been no alterations to the supportive care options for chemotherapy‐induced neutropenia.

Mecapegfilgrastim (HHPG‐19K) is a long‐acting pegylated recombinant human granulocyte‐colony stimulating factor (PEG‐rhG‐CSF) that exhibits a significantly prolonged half‐life compared to short‐acting G‐CSF.^13^, ^14^ It is administered subcutaneously once per chemotherapy cycle as a prophylactic measure. In 2018, the Chinese National Medical Products Administration (NMPA) approved mecapegfilgrastim to reduce the incidence of infection, particularly FN, in patients with non‐myeloid malignancies undergoing myelosuppressive anticancer therapy associated with a clinically significant occurrence of FN.^14^, ^15^, ^16^ Additionally, a multicenter, randomized, phase III clinical trial involving breast cancer patients undergoing myelosuppressive chemotherapy found that mecapegfilgrastim exhibited comparable efficacy and even outperformed short‐acting G‐CSF (filgrastim) in reducing the occurrence and duration of severe neutropenia.^17^ Furthermore, mecapegfilgrastim exhibited comparable tolerability and safety profiles to filgrastim. As of now, only our prospective, multicenter, non‐interventional, real‐world study has provided a midterm analysis (cutoff date: November 2020) on patients with non‐myeloid malignancies using mecapegfilgrastim for neutropenia prevention.^16^ The results demonstrated that mecapegfilgrastim was well‐tolerated in patients with various cancers. The incidence of neutropenia in primary administration was lower than that in secondary administration, and continuous administration helped maintain a lower incidence rate of neutropenia.

In gastrointestinal cancer, common chemotherapy regimens include fluorouracil, leucovorin, oxaliplatin, and irinotecan (FOLFOXIRI), fluorouracil, leucovorin, and oxaliplatin (FOLFOX), and (fluorouracil, leucovorin, oxaliplatin, and irinotecan (FOLFIRINOX). S‐1 or capecitabine‐based regimens offer more convenience due to their oral administration. However, studies have shown that patients receiving FOLFOXIRI, FOLFOX, or FOLFIRINOX may experience grade 3/4 neutropenia, with an incidence rate of up to 50%.^18^ The incidence of FN can also reach 5.4%, and there have been a case of treatment‐related FN leading to death.^19^ Compared to FOLFOXIRI, FOLFOX, or FOLFIRINOX chemotherapy, S‐1 or capecitabine‐based regimens have shown similar rates of grade 3/4 neutropenia and FN, with the highest rates reaching 41.8% and 6.9%, respectively. ^20^ However, in real‐world studies, there is a lack of research on the prophylactic use of long‐acting G‐CSF in patients with gastrointestinal cancer undergoing these chemotherapies.

Hence, we initiated this pioneering multicenter, prospective observational study to investigate the real‐world safety and effectiveness of prophylactic mecapegfilgrastim administration in patients with gastrointestinal cancer undergoing standard chemotherapy regimens. Our specific focus was on patients receiving chemotherapy based on S‐1 or capecitabine, as well as those considered at moderate/high risk for developing FN.

## 2 METHOD

### 2.1 Study design and patients

This multicenter, prospective, non‐interventional real‐world study was conducted at 66 sites across China and registered at the Chinese Clinical Trial Registry Centre (Registration No. ChiCTR2000031768). This study enrolled approximately 3000 patients with non‐myeloid malignancies. Among these, the midterm analysis, with a data cutoff in November 2020, evaluating the safety and effectiveness of prophylaxis of neutropenia with mecapegfilgrastim in 638 patients, was reported in 2021.^16^ In this article, we focused on the subset of these approximately the patients who have gastrointestinal cancer.

Patients were enrolled between May 2019 and November 2021, and all participants provided written informed consent before the initiation of the study. Conducted in alignment with the Declaration of Helsinki, this study received approval from the ethics committee at every participating center (No. 2019‐006). Data collection was facilitated through a commercial electronic data capture (EDC) system, and source document verification was carried out. The inclusion and exclusion criteria were consistent with those reported in the previous real‐world study.^16^

### 2.2 Treatment

This study was designed to observe the safety and effectiveness of mecapegfilgrastim under more complex dosing regimens in the real‐world settings. Therefore, the study was based on the prescribing choices of investigators, respects the preferences of patients, and does not specifically specify the administration timing of mecapegfilgrastim. Typically, patients were administered mecapegfilgrastim subcutaneously using either a fixed dose (6 mg) or a weight‐based dose (100 μg/kg) after 24 h of chemotherapy. It is recommended that investigators follow the National Comprehensive Cancer Network (NCCN) guidelines for hematopoietic growth factors (Version 2019.V1) for safe drug use. The complete blood count (CBC) performed on 7th to 9th day and the 14th ± 2nd day of each treatment cycle.

### 2.3 Outcomes

The primary outcome was the incidence of any adverse events following the use of mecapegfilgrastim, including clinical symptoms, abnormal vital signs, and laboratory test abnormalities, as determined by the researchers to be related to mecapegfilgrastim.

The secondary outcomes included the occurrence of grade 3/4 neutropenia and grade 4 neutropenia (ANC < 1.0 × 10^9^/L and ANC < 0.5 × 10^9^/L, respectively, as per CTCAE version 5.0) throughout all cycles of chemotherapy in patients administered mecapegfilgrastim, the incidence of FN from the initial to the fourth cycle of chemotherapy in patients receiving mecapegfilgrastim for all cycles, and the rate of infection occurrence.

### 2.4 Statistical analysis

This multicenter, prospective, non‐interventional real‐world study was conducted at 66 sites across China and registered at the Chinese Clinical Trial Registry Centre (Registration No. ChiCTR2000031768). This study enrolled approximately 3000 patients with non‐myeloid malignancies. The overall data is currently being analyzed, among which 638 patients have been reported in the previous real‐world study of prophylaxis of neutropenia with mecapegfilgrastim in patients with non‐myeloid malignancies.^16^ This article focuses on the subset of these approximately 561 patients who have gastrointestinal cancer.

Patients who received mecapegfilgrastim at least once were included in the full analysis set (FAS) for baseline and prophylactic effectiveness analysis. Participants who received mecapegfilgrastim at least once and had safety data were included in the safety set (SS) for safety analysis. Among the FAS, patients who received mecapegfilgrastim for four cycles were included in the modified FAS (mFAS) for prophylactic effectiveness analysis of continuous mecapegfilgrastim use.

Subgroup analyses were conducted to evaluate the effectiveness of mecapegfilgrastim in various chemotherapy regimens. One type of chemotherapy regimen involved S‐1/capecitabine‐based regimens, which utilized continuous oral administration of S‐1 or capecitabine for 12–15 days, either with or without other medications observed in the study. The other type consisted of FOLFOX, FOLFOXIRI, and FOLFIRINOX regimens. This type of regimen was commonly recommended for causing intermediate risk of FN in the NCCN guidelines for hematopoietic growth factors, and for intermediate/high risk of FN in the Chinese Society of Clinical Oncology (CSCO) guidelines for the standardized management of tumor chemoradiotherapy‐related neutropenia (Version 2021).^20^

The data statistical analysis was performed using SAS 9.4 statistical analysis software programming.

## 3 RESULTS

### 3.1 Patient characteristics

From May 2019 to November 26, 2021, a total of 561 patients with gastrointestinal cancer were enrolled in this study. This included 239 patients (42.6%) with colorectal cancer, 134 patients (23.9%) with esophageal cancer, 147 patients (26.2%) with gastric cancer, and 45 patients (8.0%) with pancreatic cancer, with a median age of 63 years (IQR 22–90). Among them, 375 patients (66.8%) were male, and 530 patients (94.5%) had an ECOG performance score of 0‐1. A total of 1396 chemotherapy cycles were observed with mecapegfilgrastim used for the prevention of neutropenia. Detailed baseline characteristics are shown in Table 1.

**Table 1:** Baseline characteristics.

Characteristic	All patients (FAS, *n* = 561)
Age, year	
Median (range)	63 (22–90)
Sex	
Male	375 (66.8%)
Female	186 (33.2%)
Eastern Cooperative Oncology Group performance status	
0–1	530 (94.5%)
≥2	24 (4.3%)
Missing	7 (1.2%)
Cancer type	
Colorectal cancer	239 (42.6%)
Esophageal cancer	134 (23.9%)
Gastric cancer	147 (26.2%)
Pancreatic cancer	45 (8.0%)
Prophylaxis strategy	
Primary	380 (67.7%)
Secondary	181 (32.3%)
Chemotherapy history	
Yes	367 (65.4%)
No	194 (34.6%)
History of chemotherapy‐induced febrile neutropenia or neutrophil reduction	
Yes	181 (32.3%)
No	380 (67.7%)
Radiotherapy history	
Yes	68 (12.1%)
No	491 (87.5%)
Missing	2 (0.4%)
Disease stage	
I	8 (1.4%)
II	48 (8.6%)
III	135 (24.1%)
IV	188 (33.5%)
Missing	183 (32.6%)
Baseline laboratory parameters, median (range)	
Neutrophils during the screening period (10^9^/L)	3.21 (0.03, 74.5)
Neutrophils (10^9^/L)	3 (0–75)
White blood cells (10^9^/L)	5 (1–49)
Platelets (10^9^/L)	186 (31–662)
Hemoglobin counts (g/L)	114 (65–164)

Abbreviation: FAS, full analysis set.

### 3.2 Effectiveness

In this study, out of the total 561 patients assessed, 52 patients (9.3%) occurred grade 3/4 neutropenia (ANC < 1.0 × 10^9^/L), and 15 patients (2.7%) occurred grade 4 neutropenia (ANC < 0.5 × 10^9^/L). Additionally, one patient (0.2%) experienced FN, while infections occurred in 24 patients (4.3%). Among the total of 1396 observed chemotherapy cycles, 57 cycles (4.0%) occurred grade 3/4 neutropenia, while 15 cycles (1.0%) occurred grade 4 neutropenia, and one cycle (0.1%) experienced FN.

One hundred and sixty patients underwent oral administration of S‐1 or capecitabine for 12–15 days per chemotherapy cycle. A total of 414 cycles were observed (Table 2). Among all these cycles, 6 (1.5%) experienced grade 3/4 neutropenia, while 1 (0.2%) cycle experienced grade 4 neutropenia. There were no instances of FN. The antibiotic usage period was six cycles.

**Table 2:** Summary of chemotherapy regimens analyzed in the study.

Chemotherapy regimen	Cycles
Oxaliplatin + capecitabine	120
Oxaliplatin + S‐1	115
Nab‐paclitaxel + S‐1	39
Oxaliplatin +bevacizumab + capecitabine	24
Oxaliplatin + S‐1 + PD‐1	24
Nab‐paclitaxel+ S‐1 + PD‐1	17
Oxaliplatin + capecitabine + PD‐1	15
Docetaxel + S‐1	13
Cetuximab + irinotecan + capecitabine	9
Bevacizumab + capecitabine +irinotecan	6
Oxaliplatin + capecitabine +cetuximab	5
Oxaliplatin + trastuzumab + S‐1	5
Capecitabine + others	12
S‐1 +others	10

In the oral administration regimen of S‐1 or capecitabine for 12–14 days, the timing of mecapegfilgrastim administration was analyzed (Table 3). The day chemotherapy begins was referred to as Day 1. On the day of chemotherapy initiation (Day 1), a 6 mg dose of mecapegfilgrastim was administered concurrently for two cycles, during which neutropenia did not occur. On Days 2–5, mecapegfilgrastim was administered for a total of 404 cycles, among which four cycles (1.0%) experienced grade 3/4 neutropenia, with no instances of grade 4 neutropenia. Additionally, antibiotics were used in five cycles (1.2%). Starting from Day 6, mecapegfilgrastim was administered for a total of eight cycles, during which two cycles (25.0%) experienced grade 3/4 neutropenia, 1 cycle (12.5%) experienced grade 4 neutropenia, and antibiotics were used in 1 cycle.

**Table 3:** The effectiveness of the prophylactic administration schedule of mecapegfilgrastim in capecitabine/S‐1‐based chemotherapy regimens.

	Day 1 (*n* = 2)	Day 2–Day 5 (*n* = 404)	≥ Day 6 (n = 8)
Number of cycles experiencing neutropenia			
Grade≥3	0	4 (1.0%)	2 (25.0%)
Grade 4	0	0	1 (12.5%)
Number of cycles experiencing FN	0	0	0
Number of antibiotic usage cycles	0	5 (1.2%)	1 (12.5%)

The effectiveness analysis of FOLFOX, FOLFIRI, and FOLFIRINOX regimens is presented in Table 4.

**Table 4:** Effectiveness of prophylactic use of mecapegfilgrastim in fluorouracil, leucovorin, and oxaliplatin (FOLFOX), fluorouracil, leucovorin, oxaliplatin, and irinotecan (FOLFOXIRI), and (fluorouracil, leucovorin, oxaliplatin, and irinotecan (FOLFIRINOX) chemotherapy regimens.

	FOLFIRINOX (*n* = 37)	FOLFOXIRI (*n* = 85)	FOLFOX (*n* = 167)
Number of cycles experiencing neutropenia			
Grade≥3	2 (5.4%)	8 (9.4%)	8 (4.8%)
Grade 4	1 (2.7%)	4 (4.7%)	2 (1.2%)
Number of cycles experiencing FN	0	1 (1.2%)	0
Number of antibiotic usage cycles	1 (2.7%)	6 (7.1%)	0

Abbreviation: FN, febrile neutropenia.

### 3.3 Safety

Out of all 561 patients, adverse drug reactions (ADRs) were experienced by 53 (9.4%) patients. Grade 3/4 ADRs were observed in three (0.5%) patients (Table 5). The most common ADR of any grade was an increase in white blood cells (2.9%). Grade 3/4 ADRs were only observed for anemia, decreased white blood cell count, and decreased neutrophil count, each occurring in 1 (0.2%) of the 561 patients.

**Table 5:** Treatment‐related adverse events reported in more than one patient.

Adverse events	All patients (*n* = 561)		Capecitabine/S‐1 (*n* = 116)^a^	
	Any grade	Grade ≥ 3	Any grade	Grade ≥ 3
Any event	53 (9.4%)	3 (0.5%)	4 (3.5%)	0
White blood cells increased	16 (2.9%)	0	0	0
Anemia	12 (2.1%)	1 (0.2%)	2 (1.7%)	0
Neutrophil count increased	7 (1.2%)	0	0	0
Myalgia	5 (0.9%)	0	1 (0.9%)	0
White blood cells decreased	4 (0.7%)	1 (0.2%)	0	0
Bone pain	4 (0.7%)	0	0	0
Platelet count decreased	3 (0.5%)	0	0	0
Arthralgia	3 (0.5%)	0	0	0
Fatigue	3 (0.5%)	0	0	0
Nausea	3 (0.5%)	0	0	0
Vomiting	3 (0.5%)	0	0	0
Alanine aminotransferase increased	2 (0.4%)	0	1 (0.9%)	0
Neutrophil count decreased	2 (0.4%)	1 (0.2%)	0	0

^a^ 116 patients received chemotherapy regimens containing S‐1 or capecitabine throughout all cycles.

Additionally, we analyzed ADRs in 116 patients who received chemotherapy regimens containing S‐1 or capecitabine throughout all cycles. Among these patients, ADRs of any grade were experienced by four patients (3.5%), including two patients (1.7%) with anemia, one patient (0.9%) with myalgia, and one patient (0.9%) with increased alanine aminotransferase. No grade 3/4 ADRs were observed.

## 4 DISCUSSION

To the best of our knowledge, this is the first study to present a comprehensive real‐world investigation into the prophylactic application of long‐acting G‐CSF for addressing chemotherapy‐induced neutropenia in patients with gastrointestinal cancer. We reported the effectiveness and safety of using the long‐acting G‐CSF, mecapegfilgrastim, in patients with gastrointestinal cancer who underwent S‐1/capecitabine‐based chemotherapy regimens. The timing of administration was also analyzed. Furthermore, within a real‐world setting, the study observed occurrences of severe neutropenia and instances of FN in gastrointestinal patients who received prophylactic mecapegfilgrastim after undergoing commonly used chemotherapy regimens that can cause an intermediate or high risk of FN.

S‐1/capecitabine is a commonly used chemotherapy regimens for gastrointestinal cancer. In the real‐world setting, we have observed that S‐1/capecitabine is often combined with drugs that have higher hematologic toxicity, such as oxaliplatin and albumin‐bound paclitaxel. Furthermore, there is a lack of clinical data regarding the preventive use of long‐acting G‐CSF for patients undergoing these regimens. However, in accordance with NCCN guidelines, long‐acting G‐CSFs can be administered the day after myelosuppressive chemotherapy or within 3–4 days after the chemotherapy.^12^ Our real‐world study indicated favorable timing for administering the long‐acting G‐CSF mecapegfilgrastim. When administered from Day 2 to Day 5 following S‐1/capecitabine‐based chemotherapy (404 cycles), there were no occurrences of FN or grade 4 neutropenia. When administered starting from Day 6 after chemotherapy (8 cycles), only 1 cycle (12.5%) experienced grade 4 neutropenia, and no incidences of FN were observed. We also analyzed 2 cycles of mecapegfilgrastim administered on the same day as chemotherapy initiation (Day 1), during which no FN or neutropenia occurred. Moreover, among the 116 patients who underwent chemotherapy regimens involving S‐1/capecitabine across all cycles, four patients (3.5%) experienced ADRs of any grade. These ADRs included anemia in two patients (1.7%), myalgia in one patient (0.9%), and increased alanine aminotransferase in one patient (0.9%). Notably, no ADRs of grade 3/4 were observed. This suggests that mecapegfilgrastim does not have potential adverse effects on myelosuppression or lead to unexpected hematotoxicity.^21^, ^22^ Meanwhile, most clinicians prefer to administer mecapegfilgrastim from the 2nd to the 5th day after chemotherapy initiation. However, when mecapegfilgrastim was administered starting from the 6th day and beyond after chemotherapy initiation (8 cycles), the incidence of grade ≥ 3 neutropenia was observed to be higher at 25.0%. Previous randomized trials have demonstrated a higher incidence of grade 3/4 neutropenia and FN in patients with gastrointestinal cancer who received S‐1/capecitabine. In a randomized phase III trial comparing first‐line chemotherapy for advanced gastric cancer, oxaliplatin plus S‐1 (SOX) was compared with cisplatin plus S‐1 (CS). The results revealed that grade ≥ 3 neutropenia occurred in 19.5% of patients receiving SOX compared to 41.8% of those receiving CS. Moreover, the incidence of FN was 0.9% for SOX and 6.9% for CS.^23^ Additionally, a randomized phase III trial showed that patients with locally advanced rectal cancer who received capecitabine plus irinotecan, 20% (35/178) patients experienced grade 3/4 neutropenia, and 3% (5/178) experienced FN.^24^ Therefore, administering mecapegfilgrastim during Days 2–5 of the S‐1/capecitabine treatment cycle is a preferable option.

In addition, FOLFOXIRI, FOLFOX, and FOLFIRINOX regimens, frequently mentioned in the NCCN guidelines, have been identified as common chemotherapy protocols that potentially carry a high‐risk of inducing neutropenia. In a randomized phase III trial of infusional FOLFOXIRI as first‐line treatment for metastatic colorectal cancer,^18^ a significant occurrence of grade 3/4 neutropenia (50.0%) was observed in the FOLFOXIRI group, and the incidence of FN was 5% (6/122 patients). In our real‐world study, after the administration of mecapegfilgrastim, FOLFOXIRI regimens resulted in grade 3/4 neutropenia in 8 (9.4%) of 85 cycles and FN in 1 (1.2%) of 85 cycles. Another randomized phase III study investigated the use of FOLFOX versus pegilodecakin plus FOLFOX in pancreatic cancer patients.^25^ The safety analysis revealed that in the pegilodecakin plus FOLFOX group (*n* = 283), compared to the FOLFOX group (*n* = 284), there were higher occurrences of grade 3/4 neutropenia, at 29.5% versus 22.7%. In our real‐world study, after administering mecapegfilgrastim with FOLFOX regimens, grade 3/4 neutropenia was observed in 8 (4.8%) of 167 cycles. No patients of FN were observed. In a randomized phase III trial comparing FOLFIRINOX to gemcitabine for metastatic pancreatic cancer,^19^ one patient in the FOLFIRINOX group experienced treatment‐related FN leading to death. Compared to the gemcitabine group, the FOLFIRINOX group exhibited significantly higher rates of grade 3/4 neutropenia (45.7% vs. 21.0%) and FN (5.4% vs. 1.2%). Furthermore, in a randomized phase III trial (UNICANCER‐PRODIGE 23) involving rectal cancer patients treated with neoadjuvant chemotherapy using FOLFIRINOX followed by chemoradiotherapy, grade 3/4 neutropenia and FN associated with FOLFIRINOX were observed in 17.0% (38/225) and 2.0% (5/225) of patients, respectively.^26^ In our real‐world population consisting of 37 cycles of FOLFIRINOX therapy, grade 3/4 neutropenia was observed in 2 (5.4%) cycles and no FN occurred. These findings suggest that employing mecapegfilgrastim as a prophylactic measure could be a viable option for patients with gastrointestinal cancer.

Therefore, in S‐1/capecitabine‐based chemotherapy regimens, it is recommended to administer mecapegfilgrastim from the 2nd to the 5th day after chemotherapy initiation. Clinical attention is advised for patients who receive mecapegfilgrastim starting from the 6th day and beyond after chemotherapy initiation, particularly if there is an incidence of grade 3/4 neutropenia ≥ 25.0%. In addition, although chemotherapy can provide survival benefits for gastrointestinal patients, some may experience bone marrow suppression after receiving the full dose.^2^, ^3^, ^4^, ^5^ This can lead to delays or dose reductions in treatment, potentially affecting its efficacy and causing serious adverse events associated with poor prognosis. Notably, when G‐CSF was used as prophylactic therapy to ensure patients receive an adequate dose intensity of chemotherapy, there was a significant positive impact on both response rate and OS.^27^, ^28^ Therefore, based on the results of this real‐world study, we believe that the prophylactic use of mecapegfilgrastim, which ensures patients receive full‐dose chemotherapy, may also extend survival outcomes. Further research is needed to confirm this. In addition, over the next 5 years, prophylactic G‐CSF therapy can be used not only for managing neutropenia in cancer patients but also in non‐cancer conditions, such as hyperthyroidism.^29^

This study also has some limitations. First, the patients were only observed for the treatment outcomes over 4 cycles, and long‐term follow‐up was not conducted. Additionally, the ANC data in our real‐world study was collected in accordance with routine medical diagnosis and treatment, and not through the frequent blood collection seen in randomized controlled trials. As a result, we were unable to observe the duration of ANC reduction. Future analyses can encompass a broader range of populations.

In conclusion, this study demonstrated that mecapegfilgrastim, exhibited favorable effectiveness and safety in patients with gastrointestinal cancer, including those undergoing S‐1/capecitabine‐based regimens and chemotherapy regimens associated with a high risk of FN. Our findings contribute further evidence supporting the prophylactic utilization of mecapegfilgrastim in the prevention of neutropenia in patients with gastrointestinal cancer.

## NOVELTY AND IMPACT

This is the first study to present a comprehensive real‐world investigation into the prophylactic use of long‐acting granulocyte‐colony stimulating factor (G‐CSF) for managing chemotherapy‐induced neutropenia in patients with gastrointestinal cancer. The results may provide further evidence supporting the use of mecapegfilgrastim in preventing neutropenia in these patients.

## AUTHOR CONTRIBUTIONS

**Chenyu Mao**: Conceptualization; data curation; formal analysis; supervision; writing—original draft; writing—review and editing. **Ye He**: Conceptualization; data curation; funding acquisition; supervision; writing—original draft; writing—review and editing. **Nong Xu**: Supervision; writing—original draft; writing—review and editing. **Haijiao Yan**: Supervision; writing—original draft; writing—review and editing. **Ningling Zhang**: Supervision; writing—original draft; writing—review and editing. **Gang Cheng**: Supervision; writing—original draft; writing—review and editing. **Hua Jiang**: Supervision; writing—original draft; writing—review and editing. **Minbin Chen**: Supervision; writing—original draft; writing—review and editing. **Yong Chen**: Supervision; writing—original draft; writing—review and editing. **Xiaoguang Wang**: Supervision; writing—original draft; writing—review and editing. **Yulan Gu**: Supervision; writing—original draft; writing—review and editing. **Peng Shen**: Supervision; writing—original draft; writing—review and editing. **Guifang Zhang**: Supervision; writing—original draft; writing—review and editing. **Jun Yan**: Supervision; writing—original draft; writing—review and editing. **Zhe Yang**: Supervision; writing—original draft; writing—review and editing. **Lifang Ding**: Supervision; writing—original draft; writing—review and editing. **Zhengxiang Han**: Supervision; writing—original draft; writing—review and editing. **Zhanggui Wang**: Supervision; writing—original draft; writing—review and editing. **Junqi Zhang**: Supervision; writing—original draft; writing—review and editing. **Weie Zheng**: Supervision; writing—original draft; writing—review and editing. **Jufeng Wang**: Supervision; writing—original draft; writing—review and editing. **Shukui Qin**: Conceptualization; funding acquisition; supervision; writing—original draft; writing—review and editing.

## CONFLICT OF INTEREST STATEMENT

The authors declare no conflict of interest.

## ETHICS STATEMENT

The study protocol received approval from the ethics committee at each participating center (No. 2019‐006).

## Data Availability

The data that support the findings of our study are available from the corresponding author upon reasonable request.
